# Design and Baseline Data for a Prospective Observational Study of Rivaroxaban in Patients with Venous Thromboembolism in Japan (XASSENT)

**DOI:** 10.1055/a-1664-1164

**Published:** 2021-10-07

**Authors:** Ikuo Fukuda, Atsushi Hirayama, Kazuo Kawasugi, Takao Kobayashi, Hideaki Maeda, Mashio Nakamura, Norifumi Nakanishi, Norikazu Yamada, Tsubasa Tajima, Sanghun Iwashiro, Yutaka Okayama, Toshiyuki Sunaya, Kazufumi Hirano, Takanori Hayasaki

**Affiliations:** 1 Department of Cardiovascular Surgery, Suita Tokushukai Hospital, Suita, Japan; 2 Division of Cardiology, Osaka Police Hospital, Osaka, Japan; 3 Department of Internal Medicine, Teikyo University School of Medicine, Tokyo, Japan; 4 Hamamatsu Medical Center, Hamamatsu, Japan; 5 Division of Cardiovascular Surgery, Nihon University School of Medicine, Tokyo, Japan; 6 Nakamura Medical Clinic, Kuwana, Japan; 7 Department of Cardiology, Osaka Namba Clinic, Osaka, Japan; 8 Department of Cardiology, Kuwana City Medical Center, Kuwana, Japan; 9 Medical Affairs Cardiovascular and Nephrology, Medical Affairs and Pharmacovigilance, Bayer Yakuhin, Ltd., Osaka, Japan; 10 Pharmacovigilance Monitoring and Governance, Medical Affairs and Pharmacovigilance, Bayer Yakuhin, Ltd., Osaka, Japan; 11 Statistics and Data Insights, Data Sciences and Analytics, Research and Development Japan, Bayer Yakuhin, Ltd., Osaka, Japan

**Keywords:** Japan, pulmonary embolism, rivaroxaban, venous thromboembolism, venous thrombosis

## Abstract

**Background** The efficacy and safety of rivaroxaban have been demonstrated in phase 3 trials of patients with venous thromboembolism (VTE; pulmonary embolism [PE] and deep vein thrombosis [DVT]). Data regarding rivaroxaban treatment of VTE in routine Japanese clinical practice remain limited.

**Objectives** XASSENT will evaluate rivaroxaban treatment of VTE in real-world Japanese clinical practice. We report the study design and baseline patient characteristics.

**Methods** XASSENT (NCT02558465) is an open-label, prospective observational, post-marketing surveillance cohort study in patients receiving rivaroxaban treatment for VTE. Enrolment took place between November 2015 and March 2018. XASSENT will follow patients for up to 2 years. Primary outcome variables: major bleeding and symptomatic recurrent VTE. Statistical analyses are exploratory and descriptive.

**Results** Baseline patient characteristics at June 2020 ( *n* = 2,299) are presented (58.2% female; mean age 66.7 years; mean weight 60.9 kg). The population encompasses patients with wide-ranging characteristics including older age, low weight, and renal dysfunction. Most participants (67.6%) had a history of VTE risk factors at baseline. Half of the population (50.4%) had DVT only; 41.4% had DVT with PE; 8.2% had PE only. Overall, 68.4% were inpatients and 77.1% had symptomatic VTE. Rivaroxaban was prescribed for initial treatment in 84.6% of patients and maintenance treatment in 15.4%. Most were prescribed the approved dose of rivaroxaban for initial (30 mg daily; 84.4%) or maintenance (15 mg daily; 81.9%) treatment of VTE in Japan. The most common reason for selecting non-recommended dose was ‘elderly’.

**Conclusions** Results from XASSENT will complement phase 3 trial data and inform clinical practice.

## Introduction

The prevalence of risk factors for venous thromboembolism (VTE; pulmonary embolism [PE] and deep vein thrombosis [DVT]) and the number of VTE diagnoses have increased in Japan in recent decades. 1 Direct oral anticoagulants, including edoxaban, rivaroxaban, and apixaban, became available for the treatment and prevention of recurrence of VTE in Japan in 2014 and 2015. 2 There have since been changes in the practice pattern for VTE treatment, with increasing proportions of hospitalized patients who were anticoagulated at discharge after having received direct oral anticoagulants, and decreasing proportions of these patients after receiving warfarin, according to a Japanese claims database study. 2

Rivaroxaban, an oral, direct factor Xa inhibitor, is suitable for the single-drug treatment of patients with PE or DVT. 3 4 The efficacy and safety of rivaroxaban have been evaluated in several phase 3 trials of patients with VTE ( Table 1 ). 5 6 7 8 In the large, international EINSTEIN-PE and EINSTEIN-DVT trials ( *N* = 8,282), a single-drug approach with rivaroxaban had similar efficacy to standard therapy (enoxaparin and warfarin or acenocoumarol) and was associated with a significantly lower rate of major bleeding in patients with symptomatic VTE. 8 The safety and effectiveness of rivaroxaban in routine clinical practice were then assessed in large, international registries ( Table 1 ). 9 10 Pharmacokinetic analyses had shown the exposure of rivaroxaban 15 mg administered to Japanese patients is similar to that of 20 mg to non-Japanese patients. 11 12 Moreover, target prothrombin time-international normalized ratio (PT-INR) of warfarin for VTE patients is lower in Japan (i.e., PT-INR 1.5 to 2.5 in Japan; 2.0 to 3.0 in western countries). 13 For these reasons, the smaller J-EINSTEIN-PE and J-EINSTEIN-DVT program ( *n* = 100) was performed, in which 15 or 10 mg twice daily followed by 15 mg once daily (10 mg twice daily was used only in J-EINSTEIN-DVT) were compared with Japanese standard therapy (unfractionated heparin followed by warfarin) in Japanese patients with symptomatic VTE. 5 The composite of symptomatic VTE events or asymptomatic deterioration occurred in one patient (1.3%) receiving rivaroxaban and in one patient (5.3%) receiving unfractionated heparin/warfarin (absolute risk reduction, 4.0% [95% confidence interval (CI): –2.9, 24.0]), and there was no major bleeding during study treatment. 5 Overall, the findings were consistent with those from the international EINSTEIN-PE and EINSTEIN-DVT program. 5 However, data regarding the effectiveness and safety of rivaroxaban in unselected patients with PE and/or DVT in routine clinical practice in Japan remain limited.

**Table 1:** XASSENT and phase 3 trials/clinical registries that evaluated rivaroxaban for the treatment of acute VTE globally and in Japan

Study	Design	Number of patients in rivaroxaban treatment	Patients/settings	Reference
EINSTEIN-PE and EINSTEIN-DVT	Open-label, randomized studies	2,419 patients with PE ± DVT; 1,731 patients with DVT a	Global phase 3 trials to evaluate efficacy and safety of rivaroxaban for symptomatic VTE compared with enoxaparin followed by a vitamin K antagonist (warfarin or acenocoumarol) Patients with acute, symptomatic, objectively confirmed PE and/or proximal DVT in 314 sites in 38 countries excluding Japan The mean duration of rivaroxaban treatment was 207.6 days	6 7 8
J-EINSTEIN-PE and J-EINSTEIN-DVT	Open-label, randomized studies	30 patients with PE ± DVT; 48 patients with DVT a	Japanese phase 3 trials to evaluate efficacy and safety of rivaroxaban for symptomatic VTE compared with unfractionated heparin followed by warfarin Patients with acute, symptomatic, objectively confirmed PE and/or proximal DVT in 39 sites in Japan The mean duration of rivaroxaban treatment was 195 days	5
XALIA	A prospective, non-interventional, observational study	220 patients with PE + DVT; 2,399 patients with DVT b	A study to assess the safety and effectiveness of rivaroxaban for the treatment of symptomatic DVT in routine clinical practice compared with standard anticoagulation therapy, which met a regulatory request during the assessment procedure for marketing authorization from the EMA Patients with objectively confirmed diagnosis of DVT and an indication to receive ≥3 months' anticoagulation treatment (patients with isolated PE were not eligible) in 21 countries (Austria, Belgium, Canada, Czech Republic, Denmark, France, Germany, Greece, Hungary, Israel, Italy, Moldova, the Netherlands, Norway, Portugal, Slovenia, Spain, Sweden, Switzerland, Ukraine, and the UK) The median duration of rivaroxaban treatment was 181 days. The median duration of follow-up was 239 days	9
XALIA-LEA	A prospective, non-interventional, observational study	403 patients with PE ± DVT, 882 patients with DVT b	XALIA-LEA included patients from regions different from XALIA (Indonesia, Malaysia, the Philippines, Singapore, South Korea, Taiwan, Russia, Ukraine, Jordan, Kazakhstan, Lebanon, Saudi Arabia, Algeria, Egypt, Kenya, and Mexico) Patients with objectively confirmed DVT and/or PE and an indication to receive ≥3 months anticoagulation treatment. The median duration of rivaroxaban treatment was 184 days. The median duration of follow-up was 215 days.	10
J'xactly Study	A prospective, non-interventional, observational study	419 patients with PE ± DVT; 597 patients with DVT a	A study to assess the effectiveness and safety of rivaroxaban in Japanese patients with VTE in a real-world setting Patients with acute symptomatic/asymptomatic DVT or PE ± DVT and prescribed rivaroxaban for the treatment and prevention of VTE in 152 sites in Japan The median duration of follow-up was 21.3 months	16
XASSENT	A prospective, non-interventional, observational study	1,139 (991 c ) patients with PE ± DVT; 1,159 (953 c ) patients with DVT b (June 2020)	A study to assess the safety and effectiveness of rivaroxaban in patients with VTE in routine clinical use in Japan as post-marketing surveillance Patients who were newly starting rivaroxaban for DVT or PE ± DVT (not restricted to symptomatic VTE) in 357 sites across Japan A standard observation period of 1 year and will follow patients for up to 2 years	NA

Abbreviations: DVT, deep vein thrombosis; EMA, European Medicines Agency; NA, not applicable; PE, pulmonary embolism; VTE, venous thromboembolism.

a Patients in the intention-to-treat population.

b Patients in the safety analysis.

c Patients who were prescribed rivaroxaban for initial treatment.

The Xarelto Post-Authorization Safety and Effectiveness Study in Japanese patients with Pulmonary Embolism and/or Deep Vein Thrombosis (XASSENT) is a prospective observational study that will evaluate rivaroxaban in patients with VTE in real-world Japanese clinical practice. This article describes the design of XASSENT and provides baseline data for the study population as of June 2020.

## Methods

### Study Design, Objective, and Setting

XASSENT is an open-label, single-arm, prospective, non-interventional, observational cohort study in patients for whom rivaroxaban treatment for VTE (PE and/or DVT) has been selected (ClinicalTrials.gov identifier: NCT02558465). Its objective is to assess the safety and effectiveness of rivaroxaban for patients with PE and/or DVT in routine clinical use. The study, which is being conducted at multiple medical institutions in Japan, was approved by the Japanese Ministry of Health, Labor and Welfare (MHLW) as a post-marketing surveillance and is being performed in accordance with Good Post-marketing Study Practice standards provided by the MHLW. Separate ethics approval for this post-marketing surveillance study and written informed consent to participate in the surveillance were not required under Japanese regulations, but were obtained when required by a participating center.

**Fig. 1:**
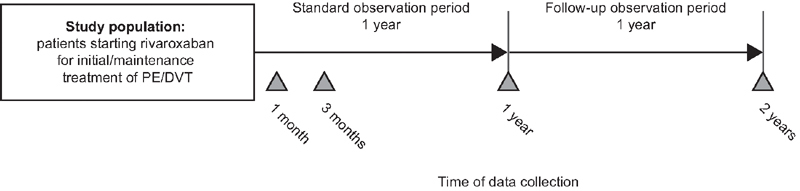
Design of XASSENT. DVT, deep vein thrombosis; PE, pulmonary embolism.

The first participant was enrolled in November 2015, after the approval of rivaroxaban for the treatment and prevention of recurrence of VTE in Japan (September 2015), and enrolment continued until March 2018. XASSENT includes a standard observation period of 1 year and will then follow patients for up to 1 year, with data collection taking place at baseline and 1 month, 3 months, 1 year and 2 years after the initiation of rivaroxaban ( Fig. 1 ). The surveillance is expected to complete on 31 March 2021.

### Participants

Consecutive patients who were newly starting rivaroxaban for the treatment and prevention of recurrence of PE/DVT (the index PE/DVT event) were registered for the study by investigators who prescribe rivaroxaban routinely. The diagnosis of PE/DVT was left to attending physicians. The recommendations for PE/DVT diagnosis methods were provided in guidelines by the Japanese Circulation Society Joint Working Group. 13 Details of diagnosis (such as diagnosis type [DVT only, DVT with PE, PE only], diagnostic method, symptoms, site of occurrence [iliac, femoral, upper extremity etc.]) were recorded on case report forms. The investigator was to have made the choice of treatment (rivaroxaban), in line with the Japanese product label, 3 before enrolling the patient.

### Treatment

In Japan, rivaroxaban is approved for the treatment and prevention of recurrence of VTE at a dose of 15 mg twice daily (30 mg daily) for the first 21 days followed by 15 mg once daily. 3 The medication is administered orally after meals. No study drug will be provided by the sponsor. If the investigator determines that rivaroxaban treatment should be discontinued or that the patient has been lost to follow-up, surveillance of the patient will be terminated. In case that rivaroxaban is discontinued, post-treatment status and adverse events (only the occurrence of bleeding events and fatal adverse events during follow-up observation period), and PE/DVT recurrence will be recorded for 3 months after rivaroxaban discontinuation, if feasible.

### Variables

Details of the variables assessed during the study are presented in Table 2 . These variables are reported by attending physicians using case report forms at enrolment and at 1 month, 3 months, 1 year and 2 years after rivaroxaban initiation. Bleeding and recurrent PE/DVT are adjudicated by attending physicians. The primary safety variable is major bleeding, defined in accordance with International Society on Thrombosis and Haemostasis criteria. 8 Specifically, major bleeding is defined as clinically overt bleeding associated with any of the following: a fatal bleeding; bleeding in a critical area or organ (e.g., intracranial, intraspinal, intraocular, pericardial, intra-articular, retroperitoneal, or intramuscular with compartment syndrome); ≥2 g/dL reduction in haemoglobin level; or transfusion of ≥2 units of whole blood or packed red blood cells. 14 The primary effectiveness variable is symptomatic recurrent VTE (a composite of non-fatal or fatal PE or DVT). The diagnosis of recurrent PE/DVT is left to attending physicians, and the details of the diagnosis are recorded on case report forms.

**Table 2:** Variables assessed during XASSENT

Variable	Information about assessments undertaken
**Primary outcome variables**	
Major bleeding	• Defined in accordance with ISTH criteria 14
Symptomatic recurrent VTE	• Composite of non-fatal or fatal PE or DVT
**Secondary outcome variables**	
All-cause mortality	–
Vascular events	• Acute coronary syndrome, ischemic stroke, transient ischemic attack, or systemic embolism
Clinically relevant non-major bleeding	• Defined as overt bleeding not meeting the ISTH criteria for major bleeding, 14 but requiring medical intervention, an unscheduled visit or telephone call, or interruption/discontinuation of rivaroxaban, or resulting in unpleasant symptoms (e.g., pain) and/or interference with daily life
Minor bleeding	• Defined as overt bleeding not meeting the definition of major or clinically relevant non-major bleeding
Asymptomatic deterioration of thrombotic burden by the end of the standard observation period	• Recorded if detected based on D-dimer levels, imaging test such as CT or CCUS
Distal and/or proximal DVT treatment outcomes	• Comparison between distal and proximal DVT outcomes
Other AEs/adverse drug reactions	• An AE is any untoward medical occurrence in a patient administered a medicinal product and which does not necessarily have a causal relationship with this treatment. The term also covers laboratory findings or results of other diagnostic procedures that are considered to be clinically relevant • An AE is considered as treatment emergent when it starts on or after the day of the first dose of study medication • An adverse drug reaction (ADR) is defined as a response to a medicinal product which is noxious and unintended (any AE judged as having a reasonable suspected causal relationship to study medication) • All AEs will be documented. For each AE, the investigator will assess and document the seriousness, duration, relationship to rivaroxaban treatment, action taken, and outcome of the event • As a post-marketing surveillance, both the investigator and the sponsor judge whether each AE is a serious adverse event (SAE), such as an AE resulting in death or life-threating AE, and whether the AE has a causal relationship to study medication. If either the investigator or the sponsor judges it to be SAE or to have a reasonable suspected causal relationship, the AE is classified as SAE or ADR.
**Patient characteristics**	
Demographics	• Date of birth or age, sex (and whether pregnant if female), blood type, inpatient/outpatient, height, weight, smoking history, alcohol use history, history of risk factors for PE/DVT
History of VTE	• Diagnosis of PE/DVT before the index PE/DVT event, date of diagnosis
Comorbidities and prior treatment	• Past medical, surgical, and interventional history (including history of hypersensitivity, renal disease [creatinine clearance, disease name], liver disease [Child-Pugh classification, disease name], cardiovascular disease, lung disease, diseases or conditions with high bleeding risk, and other comorbidities; prior treatment for the index VTE event)
**VTE diagnoses and subtypes**	
VTE diagnosis	• Diagnosis type (DVT only, DVT with PE, PE only), diagnostic method, symptoms, site of occurrence (iliac, femoral, upper extremity etc.)
Classification of clinical severity of PE	• Categories used: cardiac arrest/collapse, massive, sub-massive, non-massive, unknown
**Rivaroxaban use**	
Rivaroxaban exposure/treatment	• Purpose of the treatment (initial/maintenance treatment), daily dose, start date, reason for selecting non-recommended dose (i.e., a dose other than the approved dose), dose change status, medication adherence, reason for treatment interruption/discontinuation (if relevant), stop date
**Concomitant therapies**	
Concomitant medication	• Name of concomitant medication, route of administration, reason for administration, daily dose, start date, stop date (if relevant)
Adjunct therapy for target disease other than medication	• Therapy and treatment date
**Vital signs and laboratory findings**	
Vital signs and laboratory findings	• Recorded if performed as part of routine care • Blood pressure, pulse rate, oxygen saturation, body weight, leukocyte count, haemoglobin level, haematocrit value, platelet count, creatinine, creatinine clearance, total bilirubin, AST, ALT, ALP, LDH, albumin, D-dimer, soluble fibrin, prothrombin concentration (activity), PT, PT-INR, activated partial thromboplastin time, fibrinogen
Abnormal clinical laboratory findings associated with an AE	• Laboratory test name (e.g., D-dimer, CT, CCUS), date of measure, variables
**Visits**	• Date of visit

Abbreviations: AE, adverse event; ALP, alkaline phosphatase; ALT, alanine aminotransferase; AST, aspartate aminotransferase; CCUS, complete compression ultrasound; CT, computed tomography; DVT, deep vein thrombosis; INR, international normalized ratio; ISTH, International Society on Thrombosis and Haemostasis; LDH, lactate dehydrogenase; PE, pulmonary embolism; PT, prothrombin time; VTE, venous thromboembolism.

Secondary safety variables include all-cause mortality; vascular events (acute coronary syndrome, ischemic stroke, transient ischemic attack, or systemic embolism); clinically relevant non-major bleeding 8 ; and all other adverse events/adverse drug reactions. Secondary effectiveness variables include asymptomatic deterioration of thrombotic burden (D-dimer levels, or imaging test such as computed tomography or complete compression ultrasound) by the end of the standard observation period and distal and/or proximal DVT treatment outcomes.

Bleeding is an event of special interest and will be assessed according to the following categories: major bleeding; clinically relevant non-major bleeding; and minor bleeding. Clinically relevant non-major bleeding is defined as overt bleeding not meeting the criteria for major bleeding, 14 but requiring medical intervention, an unscheduled visit or telephone call, or interruption/discontinuation of rivaroxaban, or resulting in unpleasant symptoms (e.g., pain) and/or interference with daily life. Minor bleeding is defined as overt bleeding not meeting the definition of major or clinically relevant non-major bleeding.

Other data that will be collected relate to patient characteristics, VTE diagnoses and subtypes, comorbidities, rivaroxaban use, and concomitant therapies ( Table 2 ).

Historic data (demographic and clinical characteristics) will be collected from the patient's medical records, if available, or by interviewing the patient. If non-recommended dose (i.e., dose other than 30 mg/day for initial treatment and 15 mg/day for maintenance treatments) is selected when initiating rivaroxaban, the reason is recorded on the case report form at 1 month after the initiation of rivaroxaban. All other data required for this study will be collected during routine visits. The end of the standard observation period is 1 year after the start of rivaroxaban treatment, or earlier if rivaroxaban treatment is discontinued, or if the patient discontinues the study (e.g., is withdrawn, lost to follow-up, or dies).

The investigators will use an electronic data capture system to record data from each patient and enter these into a centralized database.

### Statistical Methods

The sample size was determined taking feasibility into account. According to the MHLW 2013 study group data, ∼40,000 patients are estimated to develop VTE (PE or DVT) in Japan each year, with patients with PE accounting for ∼40% of a Japanese population with VTE and patients with DVT accounting for the remaining ∼60%. 15 Based on the estimated recruitment rate of sites with an enrolment period of 2.5 years, the sample size was set to ≥1,250. Hemorrhage was the only important identified risk associated with rivaroxaban in the core risk management plan. Based on phase 3 studies, 5 the expected incidence of any bleeding events was 32.5%. Given these assumptions, among a sample of 1,250 patients, 406 patients would be expected to experience bleeding events during the study, which allows the capture of any bleeding events with a 95% CI of ± 2.6%. The safety analysis set will include all patients who received at least one dose of rivaroxaban and attended at least one study visit. The effectiveness analysis set will include patients who had PE/DVT diagnosis and were naïve to rivaroxaban at baseline in the safety analysis set.

Data from XASSENT will be analyzed by an independent data center. Statistical analyses are planned to be exploratory and descriptive. Data will be summarized using descriptive statistics (e.g., mean with standard deviation or median with range/interquartile range for continuous variables; frequency for categorical variables). Adverse events will be summarized using the Medical Dictionary for Regulatory Activities coding system. The number of patients with missing data will be presented as a separate category. All statistical analyses will be performed using SAS version 9.4 or higher (SAS Institute Inc., Cary, NC, USA).

For variables of interest, including the primary outcome variables, raw incidence proportion (patients with events/number of treated patients) and incidence rate (patients with events/100 patient-years) will be estimated, together with corresponding 95% CIs. Time-to-event and multivariate analyses are also planned. In addition, all analyses will be repeated with respect to relevant risk factors and Kaplan–Meier plots will show the time course up to the first event of interest.

Subgroup analyses will be conducted according to age, body weight, renal function, risk factors for VTE (including active cancer [type of cancer, metastasis, chemotherapy]), VTE subtype (PE and/or DVT, clinical severity of PE, symptoms, site/status of the thrombus), purpose of rivaroxaban administration (initial or maintenance treatment), rivaroxaban dose, treatment period (initial treatment, maintenance treatment, after discontinuation), and concomitant therapy.

## Results

Here we report demographics and baseline characteristics for the overall XASSENT population and by purpose of rivaroxaban administration (initial or maintenance treatment) (each as of June 2020). The rivaroxaban doses selected are also presented, together with reasons for choosing a dose other than the approved dose.

**Fig. 2:**
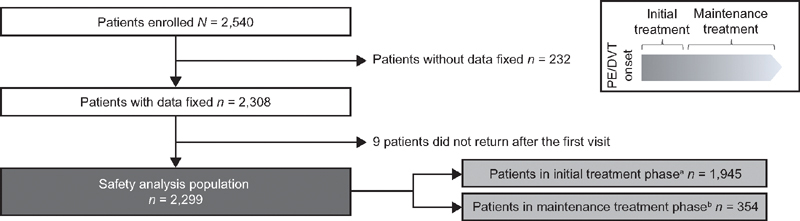
Patient flow diagram. DVT, deep vein thrombosis; PE, pulmonary embolism. ^a^ Patients who were prescribed rivaroxaban for initial treatment. ^b^ Patients who were prescribed rivaroxaban for maintenance treatment (e.g., switching from other anticoagulants, etc.).

XASSENT enrolled 2,540 patients between November 2015 and March 2018 ( Fig. 2 ). Patients were enrolled in 357 sites across Japan: 52.4% of the patients were enrolled from study sites with ≥400 beds, 32.8% from sites with 200–399 beds, 13.2% from sites with 20–199 beds, and 1.7% from sites with <20 beds. Baseline patient demographics and clinical characteristics as of June 2020 ( *n* = 2,299) are shown in Table 3 .

**Table 3:** Demographics and clinical characteristics of XASSENT participants at baseline

Characteristic	Purpose of rivaroxaban administration		Total ( *n* = 2,299)
	Initial treatment ( *n* = 1,945)	Maintenance treatment ( *n* = 354)	
Age, years	66.5 ± 15.2	67.6 ± 14.0	66.7 ± 15.0
Age category, years			
< 65	697 (35.8)	122 (34.5)	819 (35.6)
65 to <75	572 (29.4)	98 (27.7)	670 (29.1)
≥75	676 (34.8)	134 (37.9)	810 (35.2)
Female	1,115 (57.3)	223 (63.0)	1,338 (58.2)
Body weight, kg	61.3 ± 13.9	58.7 ± 13.7	60.9 ± 13.9
Body weight category, kg			
> 50	1,459 (75.0)	252 (71.2)	1,711 (74.4)
≤50	418 (21.5)	90 (25.4)	508 (22.1)
Unknown	68 (3.5)	12 (3.4)	80 (3.5)
BMI a	24.0 ± 4.2	23.5 ± 4.4	24.0 ± 4.2
Creatinine clearance, mL/min	83.8 ± 37.1	79.0 ± 35.1	83.0 ± 36.8
Creatinine clearance category, mL/min			
< 30	9 (0.5)	2 (0.6)	11 (0.5)
30 to <50	273 (14.0)	60 (16.9)	333 (14.5)
50 to <80	738 (37.9)	141 (39.8)	879 (38.2)
≥80	851 (43.8)	137 (38.7)	988 (43.0)
Unknown	74 (3.8)	14 (4.0)	88 (3.8)
Inpatients	1,417 (72.9)	156 (44.1)	1,573 (68.4)
Outpatients	528 (27.1)	198 (55.9)	726 (31.6)
VTE diagnosis			
DVT only	953 (49.0)	206 (58.2)	1,159 (50.4)
Isolated distal DVT	323 (16.6)	65 (18.4)	388 (16.9)
DVT other than isolated distal	611 (31.4)	111 (31.4)	722 (31.4)
Unidentifiable	0 (0.0)	3 (0.8)	3 (0.1)
Unknown	19 (1.0)	27 (7.6)	46 (2.0)
PE only	150 (7.7)	38 (10.7)	188 (8.2)
PE with DVT	841 (43.2)	110 (31.1)	951 (41.4)
Clinical severity of PE			
Cardiac arrest/collapse	9 (0.5)	5 (1.4)	14 (0.6)
Massive	59 (3.0)	7 (2.0)	66 (2.9)
Sub-massive	306 (15.7)	37 (10.5)	343 (14.9)
Non-massive	586 (30.1)	76 (21.5)	662 (28.8)
Unknown	31 (1.6)	23 (6.5)	54 (2.3)
Other	1 (0.1)	0 (0.0)	1 (<0.1)
VTE symptoms			
Symptomatic PE/DVT	1,551 (79.7)	221 (62.4)	1,772 (77.1)
Asymptomatic PE/DVT	393 (20.2)	133 (37.6)	526 (22.9)
History of PE/DVT	183 (9.4)	98 (27.7)	281 (12.2)
History of risk factors for VTE b	1,320 (67.9)	233 (65.8)	1,553 (67.6)
Immobilization within 3 months	366 (18.8)	61 (17.2)	427 (18.6)
Surgery/injury within 3 months	379 (19.5)	64 (18.1)	443 (19.3)
Obesity	355 (18.3)	44 (12.4)	399 (17.4)
Active cancer	312 (16.0)	74 (20.9)	386 (16.8)
Comorbidities other than risk factors for PE/DVT			
Renal disease	173 (8.9)	39 (11.0)	212 (9.2)
Liver disease	128 (6.6)	37 (10.5)	165 (7.2)
Cardiovascular disease	900 (46.3)	174 (49.2)	1,074 (46.7)
Hypertension	783 (40.3)	134 (37.9)	917 (39.9)
Atrial fibrillation	78 (4.0)	17 (4.8)	95 (4.1)
Lung disease	193 (9.9)	37 (10.5)	230 (10.0)
Diseases or conditions with high risk of bleeding c	306 (15.7)	63 (17.8)	369 (16.1)
Use of antiplatelet drugs d	154 (7.9)	42 (11.9)	196 (8.5)
Anticoagulation therapy other than rivaroxaban			
Anticoagulation for the index PE/DVT (initial treatment only)	441 (22.7)	—	—
Anticoagulation within 3 months before the initiation of rivaroxaban (maintenance treatment only)	—	228 (64.4)	—
Unfractionated heparin	377 (19.4)	97 (27.4)	—
Fondaparinux	2 (0.1)	0 (0.00)	—
Warfarin	44 (2.3)	111 (31.4)	—
Other	129 (6.6)	64 (18.1)	—
Use of inferior vena cava filter	116 (6.0)	—	—

Abbreviations: BMI, body mass index; DVT, deep vein thrombosis; PE, pulmonary embolism; VTE, venous thromboembolism.

Data cut-off: June 2020. Data are presented as mean ± standard deviation or as *n* (%).

a BMI missing for 191 patients (163 for initial treatment and 28 for maintenance treatment).

b Risk factors reported in ≥10% of total patients are listed.

c Includes haemostasis or coagulation disorders, congenital or acquired haemorrhagic disorders, uncontrollable severe hypertension, vascular retinopathy, active cancer, active ulcerative gastrointestinal disorders, short days after the onset of gastrointestinal ulcers, short days after the onset of intracranial hemorrhage, vascular abnormalities in the spinal cord or brain, short days after cerebral spinal cord or eye surgery, and history of bronchiectasis or pulmonary hemorrhage.

d During observational periods.

Overall, 58.2% of the XASSENT participants are female. At baseline, the mean age was 66.7 years, the mean body weight was 60.9 kg, and the mean creatinine clearance was 83.0 mL/min ( Table 3 ). However, the study population encompasses patients with a wide range of characteristics, including elderly patients, individuals with a low body weight, and patients with renal dysfunction (as assessed by creatinine clearance) ( Table 3 ). Most of the participants (67.6%) had a history of risk factors for VTE at baseline, with active cancer reported for 386 patients (16.8%) ( Table 3 ). Cardiopulmonary disease was reported as a risk factor for VTE for 140 patients (6.1%). Half of the XASSENT population (50.4%) had a diagnosis of DVT only, with 16.9% overall having isolated distal DVT; 41.4% had DVT with PE, and 8.2% had PE only (of varying severity) ( Table 3 ). Approximately two-thirds of the participants (68.4%) were inpatients. Most patients (77.1%) had symptomatic VTE, but patients with asymptomatic VTE were also represented ( Table 3 ).

**Fig. 3:**
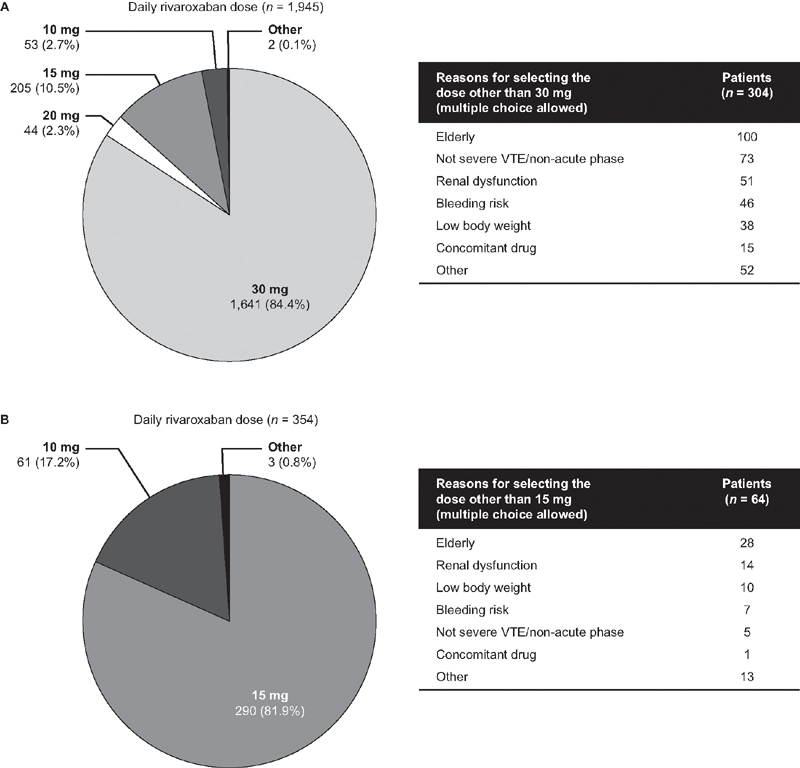
Rivaroxaban doses selected for (A) initial treatment and (B) maintenance treatment in XASSENT participants, with reasons for selecting non-recommended dose (i.e., a dose other than the approved dose). Data are presented as *n* (%) or as *n* . VTE, venous thromboembolism. Note: The reasons for the dose selection described by attending physicians were categorized and tabulated. Bleeding risk was a judgement by attending physicians. Reasons for a patient who received 15 mg in the initial treatment group are missing.

The purpose of rivaroxaban administration was initial treatment in 1,945 patients (84.6%) and maintenance treatment in 354 patients (15.4%) ( Table 3 and Fig. 3 ). The mean creatinine clearance was 83.8 mL/min in patients receiving rivaroxaban as initial treatment, while it was 79.0 mL/min in those receiving rivaroxaban as maintenance treatment ( Table 3 ). Among patients in the initial treatment group, 72.9% were inpatients, 79.7% had symptomatic VTE, and 49.0% had DVT only. Those were respectively 44.1%, 62.4%, and 58.2% among those in the maintenance treatment group ( Table 3 ). In the maintenance treatment group, 7.6% of patients had DVT only with an unknown site ( Table 3 ). Obesity and a history of VTE were reported in 18.3% and 9.4% of patients in the initial treatment group, while those were 12.4% and 27.7% in the maintenance treatment group ( Table 3 ). In the initial treatment group, 22.7% of patients received anticoagulation other than rivaroxaban for the index PE/DVT (mainly unfractionated heparin). Inferior vena cava filter was placed in 6.0% of patients ( Table 3 ), and low proportions of patients underwent thrombolysis, thrombectomy, or catheter-assisted thrombus removal (fragmentation or aspiration thrombectomy) (4.4%, 0.1%, and 0.8%, respectively). In the maintenance treatment group, 64.4% of patients received unfractionated heparin, warfarin, and other anticoagulants (except fondaparinux) in the 3 months before the initiation of rivaroxaban ( Table 3 ).

Around 80% of patients were prescribed the dose of rivaroxaban approved for the initial or maintenance treatment of VTE in Japan ( Fig. 3 ). The most common reason for selecting non-recommended dose (i.e., other than 30 mg/day in initial treatment or 15 mg/day in maintenance treatment) was that the patient was elderly ( Fig. 3 ). Other reasons included non-severe/non-acute VTE, the presence of renal dysfunction, bleeding risk (judged by attending physicians), low body weight, and the use of a concomitant drug ( Fig. 3 ).

## Discussion

XASSENT is evaluating the safety and effectiveness of rivaroxaban in patients with VTE in routine Japanese clinical practice. These results will provide real-world evidence that may complement data from the phase 3 J-EINSTEIN-PE and J-EINSTEIN-DVT trials. 5 In total, 2,540 patients with a broad range of characteristics, VTE subtypes, and comorbidities have been enrolled and will be followed for up to 2 years. XASSENT will provide incidence data for multiple variables, with a focus on bleeding and recurrent VTE events. The design of the study allows the collection of detailed information related to exposure, minimizes recall bias, and provides information on the timing of events relative to rivaroxaban administration. Exploratory subgroup analyses are planned to provide information for patient groups of interest.

The J'xactly Study 16 is another prospective observational study conducted in 1,039 Japanese patients with VTE who were prescribed rivaroxaban ( Table 1 ). Baseline characteristics were generally comparable between the two studies, although the J'xactly Study included higher proportions of outpatients (41.5%), patients with DVT only (58.8%), and patients with creatinine clearance <50 mL/min (22.4%). 16 In the J'xactly Study, the incidence of recurrence or aggravation of symptomatic VTE was 2.6% per patient-year and the incidence of International Society on Thrombosis and Haemostasis major bleeding was 2.9% per patient-year. 16 A single-drug approach with rivaroxaban was shown to be a valuable treatment for a wide range of patients with VTE in Japanese clinical practice. 16 However, more real-world data are required to support the single-drug approach with rivaroxaban for Japanese patients with VTE. In the J'xactly Study, 65.6% of patients received an initial rivaroxaban dose of 30 mg daily included in the Japanese product label. 16 In contrast, 84.4% of XASSENT participants received an initial daily rivaroxaban dose of 30 mg, with lower proportions receiving other doses owing to patient-related factors (e.g., older age).

Safety and effectiveness data from patients who were not well represented in phase 3 trials or the XALIA non-interventional study of rivaroxaban, such as patients with asymptomatic VTE ( Table 1 ), 5 8 9 10 will help to inform unmet needs related to the management of VTE. XASSENT will provide further evidence regarding patients with VTE in Japan, particularly for patients who are elderly or who have a low body weight, renal dysfunction, or active cancer. 5 8 17 For example, 17% of XASSENT participants had active cancer at baseline, compared with 6% of the rivaroxaban groups in the EINSTEIN-PE and EINSTEIN-DVT trials. 17 Patients with cancer and venous thrombosis are more likely to develop recurrent thromboembolism (∼4 times) and major bleeding (∼2 times) during anticoagulant treatment than those without cancer. 18

XASSENT is one of the largest real-world observational studies of VTE treatment and prevention in Japan. It will add to evidence from other Japanese real-world studies of VTE management, such as the Japan VTE Treatment Registry (JAVA) 19 or COMMAND VTE Registry, 20 which mainly enrolled patients before the introduction of direct oral anticoagulants for VTE in Japan, and the Edoxaban Treatment in routine cliNical prActice in patients with Venous ThromboEmbolism – Japan (ETNA-VTE-Japan) study of edoxaban. 21

Open-label, single-arm observational studies such as XASSENT have inherent limitations, including the possibility of selection bias (e.g., arising from the investigators' choices in routine clinical practice), confounding variables (e.g., dose of rivaroxaban used chosen at discretion of attending physicians), loss of patients to follow-up that may result in underestimation of incidence of clinical events evaluated by attending physicians, and the lack of mandatory laboratory tests. Furthermore, no formal diagnosis methods or criteria for diagnosis of VTE are specified in this study. However, the recommendations for the diagnosis methods were provided in guidelines, 13 and the diagnosis methods used and clinical presentations (e.g., symptoms, site of occurrence) are recorded on case report forms by attending physicians. The study is not powered to evaluate rare events.

In conclusion, XASSENT has enrolled 2,540 patients with VTE who are being treated with rivaroxaban and is one of the largest real-world observational studies of VTE management in Japan. XASSENT participants have a broad range of characteristics and comorbidities, representing real-world patients with VTE. XASSENT will provide real-world information on the safety and effectiveness of rivaroxaban for VTE treatment in routine Japanese care, to complement data from phase 3 trials and inform clinical practice.
